# Effects of SGLT-2 inhibitors on adipose tissue distribution in patients with type 2 diabetes mellitus: a systematic review and meta-analysis of randomized controlled trials

**DOI:** 10.1186/s13098-023-01085-y

**Published:** 2023-05-31

**Authors:** Xianghong Wang, Niujian Wu, Chuanchuan Sun, Donghua Jin, Hongyun Lu

**Affiliations:** 1 Department of Endocrinology and Metabolism, Zhuhai Hospital Affiliated with Jinan University (Zhuhai People’s Hospital), Zhuhai, China; 2 Department of Nephrology, The First Affiliated Hospital of Jinan University, Guangzhou, China; 3 Department of Intensive Care Unit, The Third People’s Hospital of Zhengzhou, Henan, China

**Keywords:** SGLT2 inhibitors, Adipose tissue, Type 2 diabetes, Meta-analysis

## Abstract

**Objective:**

Sodium-glucose cotransporter-2 (SGLT-2) inhibitors therapies were reported to affect adipose tissue distribution. However, the available evidence about the effect of SGLT-2 inhibitor on adipose tissue is contradictory. We conducted a systematic review and meta-analysis of randomized controlled trials (RCTs) to evaluate the effect of SGLT-2 inhibitors on adipose tissue distribution in patients with type 2 diabetes mellitus (T2DM).

**Methods:**

RCTs on SGLT-2 inhibitors on adipose distribution affect in patients with T2DM published in full-text journal databases such as PubMed, Embase, Cochrane Library, and ClinicalTrials.gov databases were searched. The fixed or random effect model was used for meta-analysis, the I^2^ test was used to evaluate the heterogeneity between studies, and the sensitivity analysis and subgroup analysis were used to explore the source of heterogeneity. Funnel chart and Begg’s test were used to estimate publication bias.

**Results:**

Overall, 18 RCTs involving 1063 subjects were evaluated. Compared with placebo or other hypoglycemic drugs, SGLT-2 inhibitors significantly reduced visceral adipose tissue (standard mean deviation [SMD] = − 1.42, 95% confidence interval [CI] [− 2.02, − 0.82], I^2^ = 94%, *p* < 0.0001), subcutaneous adipose tissue (SMD = − 1.21, 95% CI [− 1.99, − 0.42], I^2^ = 93%, *p* = 0.003), ectopic liver adipose tissue (SMD = − 0.70, 95% CI [− 1.20, − 0.20], I^2^ = 73%,* p* = 0.006). In addition, body weight (mean deviation [MD] = − 2.60, 95% CI [− 3.30, − 1.89], I^2^ = 95%, *p* < 0.0001), waist circumference (MD = − 3.65, 95% CI [− 4.10, − 3.21], I^2^ = 0%, *p* < 0.0001), and body mass index (BMI) (MD = − 0.81, 95% CI [− 0.91, − 0.71], I^2^ = 23%, *p* < 0.0001) were significantly decreased. However, epicardial fat tissue showed an insignificant reduction (SMD = 0.03, 95% CI [− 0.52, 0.58], I^2^ = 69%, *p* = 0.71). Subgroup analysis revealed that appropriate treatment duration (16 – 40 weeks) or young patients with nonalcoholic fatty liver disease (NAFLD) and obesity were the decisive factors for SGLT-2 inhibitors to effectively reduce visceral and subcutaneous adipose tissues.

**Conclusions:**

Our meta-analysis provides evidence that in patients with T2DM, SGLT-2 inhibitors significantly reduce visceral adipose tissue, subcutaneous adipose tissue, and ectopic liver fat, especially in young T2DM patients with NAFLD and high BMI. Appropriate dosing time (16–40 weeks) may have a more significant and stable beneficial effect on VAT and SAT reduction.

**Supplementary Information:**

The online version contains supplementary material available at 10.1186/s13098-023-01085-y.

## Background

Diabetes has become increasingly prevalent and caused a global economic burden, especially in developing countries [1]. Patients with type 2 diabetes mellitus (T2DM) have a high risk of cardiovascular disease, which can aggravate the progression of atherosclerosis and heart failure and eventually result in disability or death [2, 3]. Abnormal fat distribution is very common in patients with T2DM. The adipose tissue deposited in the body can be divided into visceral adipose tissue (VAT), subcutaneous adipose tissue (SAT), and ectopic adipose tissue (fat deposition in the liver, epicardium, pancreas, and skeletal muscles). Obese patients with T2DM have the characteristic ectopic fat and VAT deposition [4]. VAT and SAT are independent risk markers of cardiovascular incidence and associated mortality [5, 6]. A high percentage of VAT will increase the risk of T2DM-associated cardiovascular disease. Ectopic fat deposition, such as fat deposited in the liver and epicardial adipose tissue (EAT), is also associated with common cardiovascular metabolic complications of T2DM [7]. This means that fat accumulation is an important clinical problem, especially in obese patients with T2DM. Reduction of visceral fat and ectopic adipose tissue may reduce in improving the risk of T2DM metabolic syndrome and cardiovascular disease.

Sodium-glucose cotransporter-2 (SGLT-2) inhibitors are a new class of antidiabetic drugs. They act on sodium glucose cotransporters in the renal tubules and inhibit glucose reabsorption in these tubules, consequently promoting glucose excretion in urine to reduce plasma glucose levels [8]. SGLT-2 inhibitors also reduce the risk of major adverse cardiovascular events. Recent large-scale clinical trials such as the EMPA-REG Study [9], CANVAS Program [10], Declare-TIMI 58 [11] have demonstrated that different types of SGLT-2 inhibitors significantly reduce the cardiovascular incidence/mortality and heart failure–related hospitalization rates in patients with T2DM and confirmed cardiovascular disease. SGLT-2 inhibitors can also reduce body weight and obesity index [12].

Recently, Liu et al. [13]. conducted a excellent meta-analysis that demonstrated the significant reduction of VAT and SAT in patients with type 2 diabetes following the administration of SGLT-2 inhibitors. However, it is imperative to note that adipose tissue comprises not only these two types of tissue, but also includes ectopic adipose tissue such as liver fat and epicardial adipose tissue. The impact of SGLT-2 inhibitors on these types of adipose tissue in patients with type 2 diabetes remains a controversial topic. Moreover, previous studies lacked detailed subgroup analysis to explore potential influencing factors of SGLT-2 inhibitors on adipose tissue. Furthermore, some recent clinical studies have emerged. Hence, it is necessary to conduct a new RCT meta-analysis to study the effects of SGLT-2 inhibitors on adipose tissue in adults with type 2 diabetes. We also performed a comprehensive subgroup analysis to explore the subgroups of patients who may benefit more from treatment with SGLT-2 inhibitors.

## Methods

For the systematic review and meta-analysis, we followed the Preferred Reporting Items for Systematic Reviews and Meta-Analyses (PRISMA) guidelines [14]. The registered application of the systematic evaluation scheme has been registered in the PROSPERO database (registration number: CRD42023401163).

### Search strategy

We performed a literature search on four major medical databases, namely, PubMed, Cochrane Library, Embase, and ClinicalTrials.gov, from inception of the database to January 2, 2023. The search terms are as follows: (Sodium–Glucose Transporter 2 Inhibitors OR SGLT-2 OR dapaglilozin OR canagliflozin OR tofoglifloxin OR empagglifloxin OR ertuglifloxin OR ipraglifloxin OR lusoglifloxin OR remogliflozin OR sergliflozin) and (adipose tissue OR visceral fat OR visceral adipose OR ectopic fat OR ectopic adipose OR subcutaneous adipose OR subcutaneous fat), AND (randomized controlled trial [Publication Type]). Publication date and language restrictions were not applied, and the reference list of the selected articles was screened to supplement the search strategy.

### Selection criteria

The RCTs included in this study had to meet the following inclusion criteria: (1) The study design was an RCT, (2) the subject had a clinical diagnosis of T2DM, (3) the intervention drug was an SGLT-2 inhibitor, and (4) the study reported results for changes in the adipose tissue of the SGLT-2 group relative to the control group. That is, the study should have reported the mean value and standard deviation (SD) before and after treatment (or provided adequate data to calculate these values).

Studies that met the following exclusion criteria were removed during screening: (1) The study was a summary, brief report, or meeting summary or included animal and cell experiments, (2) the study with inaccessible full text or incomplete data, and (3) the study was a duplicate publication or included studies with similar information. For studies evaluating multi-dose SGLT-2 inhibitors, only the highest dose group was included in the meta-analysis. The same criteria were used in the evaluation of SGLT-2 inhibitors at different durations, including only the group with the longest follow-up time.

Based on the inclusion and exclusion criteria, the selection of articles was performed independently by two reviewers (Xianghong Wang and Niujian Wu), who reviewed the title and abstract of each retrieved article. In cases of any uncertainty about the qualification of a study, a third researcher (Hongyun Lu) read the full text and arrived at a decision. All studies have reached a consensus.

### Data extraction

The two reviewers independently extracted data from RCTs that met the criteria and the Cochrane Reviewer’s Handbook. All the authors discussed the results in the event of discrepancies. The extracted data included information of the participants’ baseline characteristics (including first author, country of origin, age, sex, body mass index), publication year, sample size, intervention measures (the SGLT-2 inhibitor used and control group medication type and daily dose), duration of medication, examination method for main outcome indicators, mean change and standard deviation (SD) of outcomes from baseline to the end in main parameters (VAT, SAT, and ectopic adipose tissue), and other body components (body weight, BMI, and waist circumference). In case the article contained other data that could be converted into mean and SD, such as standard error (SE), 95% confidence interval (CI), and median and interquartile range, we used the following formula to convert the data: SD_E,change_ = √(SD^2^_E,baseline_ + SD^2^_E,final_ − [2 × Corr × SD_E,baseline_ × SD_E,final_] [15], SD = SE × √n [15], SD = √n (upper limit − lower limit)/3.92 [15]; in case the studies reported interquartile range, we used the following formula to convert the data: mean ≈ (0.7 + 0.39/n)/(q1 + q3)/2 + (0.3 − 0.39/n)m [16], SD ≈ (q3 − q1)/(2Ф − 1 [0.75 n − 0.125]/[n + 0.25]) [17]. For studies that did not report data, we sent an email to the corresponding author requesting for further information and used the formula described in the Cochrane’s manual for conversion, when needed.

### Quality assessment

The two researchers evaluated the risk of bias in each qualified study using the tools of Cochrane Collaboration’s tool [18], the risk of bias includes seven potential sources of bias: allocation sequence generation, allocation concealment, participant blindness, result evaluation blindness, incomplete result data, selective result report and other possible bias. According to the recommendations of the Cochran Manual, the risk of bias in each study was assessed as “low risk,” “high risk,” or “unclear risk.”

### Data synthesis and analysis

The data were analysed using Review Manager 5.4 (The Cochrane collaboration, Oxford, England) and Stata 12.0 (StataCorp, Texas, USA). When different measurement methods or units were used for the research results, we used the standard mean deviation (SMD) and 95% CI values to synthesize the effect size. Otherwise, we used the mean deviation (MD) and 95% CI values. The I^2^ value was used to evaluate the heterogeneity between studies. I^2^ ≥ 50% or the corresponding *p* value (*p* < 0.05) was considered to have significant heterogeneity in the results between studies; in such cases, we used a random-effects model. I^2^ < 50% or the corresponding *p* value (*p* > 0.05) was considered to have no significant heterogeneity in the results between studies; in such cases, we used a fixed-effects model. If the test level α is set as 0.05, less than 0.05 indicates that the results are statistically significant. Furthermore, we conducted subgroup analyses and sensitivity analyses to determine the source of the heterogeneity. Finally, STATA 12.0 was used to construct a funnel map and perform Egger’s test to determine the presence of publication bias. *p* < 0.05 was considered statistically significant.

## Results

### Search results and study characteristics

**Fig. 1:**
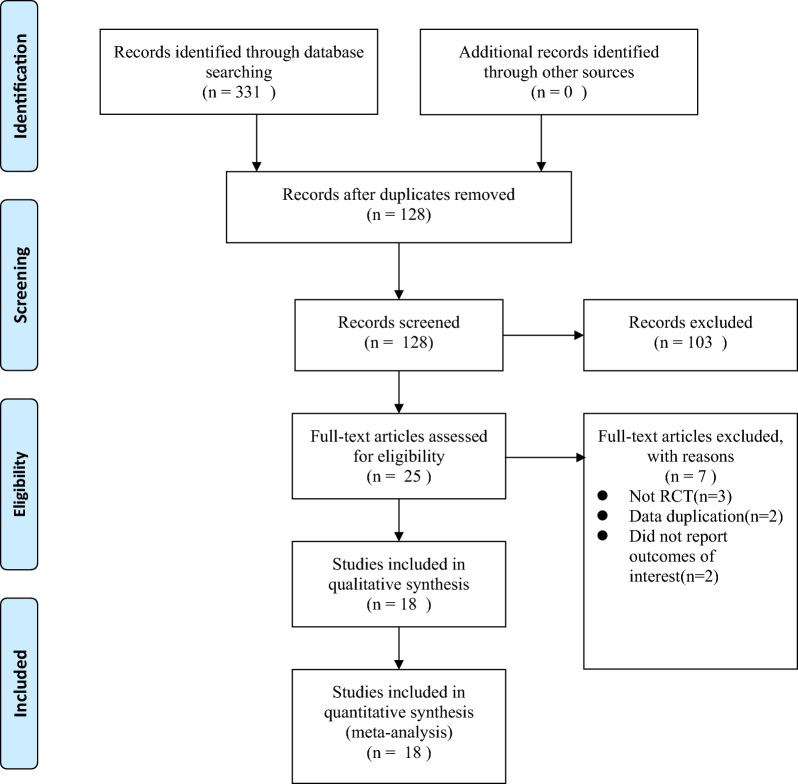
Study flow diagram

The initial search revealed 331 relevant articles; 203 duplicate articles were excluded, and the remaining 128 were screened based on the title and abstract. After preliminary screening, 25 articles had to be evaluated based on the full text. Finally, 18 RCTs were included. The flow chart of study selection is shown in Fig. 1.

Among the 18 RCTs (n = 1063 patients), 16 reported VAT [19–34], 9 reported SAT [20–24, 30, 32–34], 3 reported EAT [19, 35, 36], and 5 reported liver fat [19, 23, 24, 31, 34]. Table 1 summarizes data of the main features of the included RCTs. All studies were published during 2017–2022. All participants had a clinical diagnosis of T2DM, and 7 articles assessed participants with NAFLD [23, 24, 28, 29, 31, 33, 34]. The duration of the RCTs ranged from 8 to 48 weeks. Interventions of SGLT-2 inhibitors included dapagliflozin [21, 22, 24, 29, 30, 34, 35], empagliflozin [19, 27, 28, 31, 36], ipragliflozin [20, 23, 25, 32, 33] and canagliflozin [26]. Nine RCTs compared SGLT-2 inhibitors with placebo and 9 studies compared the inhibitors with other antidiabetic drugs, namely, sitagliptin, semaglutide, pioglitazone, glimepiride, or metformin.

**Table 1:** Characteristics of included RCTs

Study ID	Country	Sample size, n	Intervention	Comparator	Co‐intervention	Age(y)	Duration	Population	Baseline BMI (kg/m^2^)	Outcome evaluation	Included outcome
B. Gaborit 2021	France	26/25	Empaglifozin 10 mg qd	Placebo	None	57.0 ± 10.1/58.6 ± 9.2	12 weeks	T2DM	33.7 ± 3.8/34.7 ± 7.1	MRI	VAT, EAT, liver fat
Masaya Koshizaka 2021	Japan	15/14	Ipragliflozin 50 mg qd	Metformin 500 mg qd	None	68.7 ± 2.4/69.4 ± 2.9	24 weeks	T2DM	26.8 ± 3.8/27.1 ± 3.9	CT	VAT
Eugene Han 2020	Korea	29/15	Ipragliflozin 50 mg qd	Placebo	Metformin + pioglitazone	52.5 ± 10.3/56.7 ± 11.8	24 weeks	T2DM and NAFLD	30.4 ± 5.4/30.2 ± 2.5	CT	VAT, SAT, liver fat
Alexander J.M. Brown 2020	United Kingdom	31/34	Dapaglilflozin 10 mg qd	Placebo	None	64.3 ± 7.0/66.7 ± 6.6	48 weeks	T2DM	32.3 ± 4.7/32.6 ± 4.2	MRI	VAT, SAT
Gianluca Iacobellis 2020	USA	42/42	Dapagliflozin 10 mg qd	Placebo	Metformin	52 ± 9/51 ± 11	24 weeks	T2DM	36.6 ± 7.8/34.7 ± 6	Echocardiography	EAT
Tomoe Kinoshita 2020	Japan	32/33	Dapagliflozin 5 mg qd	Glimepiride 1 mg qd	None	58.7 ± 1.6/58.0 ± 2.3	28 weeks	T2DM and NAFLD	29.5 ± 0.8/28.4 ± 0.7	CT	VAT
Rory J. McCrimmon 2020	United Kingdom	90/88	Canagliflozin 300 mg qd	Semaglutide 1.0 mg qw	None	58.6 ± 10.1/57.8 ± 9.9	52 weeks	T2DM	32.3 ± 5.5/32.6 ± 6.4	DXA	VAT
Shintaro Sakurai 2020	Japan	31/18	Empagliflozin 10 mg qd	Standard treatment without SGLT2 inhibitors	None	58.6 ± 12.9/58.6 ± 12.2	12 weeks	T2DM	27.2 ± 5.7/28.4 ± 6.6	BIA	VAT
Aino Latva-Rasku 2019	Finland	15/16	Dapagliflozin 10 mg qd	Placebo	None	60 ± 7.4/62 ± 8.4	8 weeks	T2DM	32.1 ± 3.9/31.7 ± 5.0	MRI	VAT, SAT
Daisuke Ito 2017	Japan	32/34	Ipragliflozin 50 mg qd	Pioglitazone 30 mg qd	None	57.3 ± 12.1/59.1 ± 9.8	24 weeks	T2DM and NAFLD	30.7 ± 5.0/29.9 ± 6.2	CT	VAT, SAT
Yukihiro Bando 2017	Japan	37/21	Ipragliflozin 50 mg qd	Standard treatment without SGLT2 inhibitors	None	54.8 ± 9.3/55.4 ± 7.5	12 weeks	T2DM	27.8 ± 3.9/27.3 ± 3.1	CT	VAT, SCT
Takashi Shibuya 2017	Japan	16/16	Luseogliflozin 2.5 mg qd	Metformin 1500 mg qd	None	53.5 ± 12.2/59.6 ± 10.6	24 weeks	T2DM and NAFLD	27.6 ± 2.0/28.1 ± 5.8	CT	VFA
Masanori Shimizu 2019	Japan	33/24	Dapagliflozin 5 mg qd	Standard treatment without SGLT2 inhibitors	None	56.2 ± 11.5/57.1 ± 13.8	24 weeks	T2DM and NAFLD	27.6 ± 4.7/28.7 ± 3.5	BIA	VAT, SAT, liver fat
Shigenori Hiruma 2021	Japan	21/21	Empagliflozin 10 mg qd	Sitagliptin 100 mg qd	None	52.8 ± 9.7/47.8 ± 11.5	12 weeks	T2DM	28.6 ± 4.8/30.0 ± 5.0	MRI	EAT
Jan W. Eriksson 2018	Sweden	19/19	Dapagliflozin 10 mg qd	Placebo	None	65.0 ± 6.5/65.6 ± 6.1	12 weeks	T2DM and NAFLD	30.5 ± 2.8/30.3 ± 3.1	MRI	VAT, SAT, liver fat
Kayo Horibe 2022	Japan	26/24	Dapagliflozin 5 mg qd	Placebo	OAD	59.7 ± 12.0/62.3 ± 6.5	24 weeks	T2DM	28.0 ± 4.0/27.6 ± 3.8	MRI	VAT, SAT
Hideka Inoue 2019	Japan	24/24	Ipragliflozin 50 mg qd	Placebo	Insulin	60.5 ± 9.8/60.8 ± 12.1	24 weeks	T2DM	27.9 ± 4.0/27.7 ± 4.5	MRI	VAT, SAT
Haleh Chehrehgosha 2021	Iran	35/37	Empagliflflozin 10 mg qd	Placebo	None	50.5 ± 8.4/51.8 ± 7.8	24 weeks	T2DM and NAFLD	30.9 ± 3.3/30.2 ± 4.4	DXA	VAT, liver fat

*VAT* visceral adipose tissue, *SAT* subcutaneous adipose tissue, *OAD* oral anti-diabetic drugs, *SGLT2* sodium–glucose transporter 2, *NAFLD* non‐alcoholic fatty liver disease, *T2DM* type 2 diabetes mellitus, *DXA* dual-energy x-ray absorptiometry, *BIA* bioelectrical impedance analysis, *CT* computed tomography, *MRI* magnetic resonance imaging

### Risk of bias in included studies

**Fig. 2:**
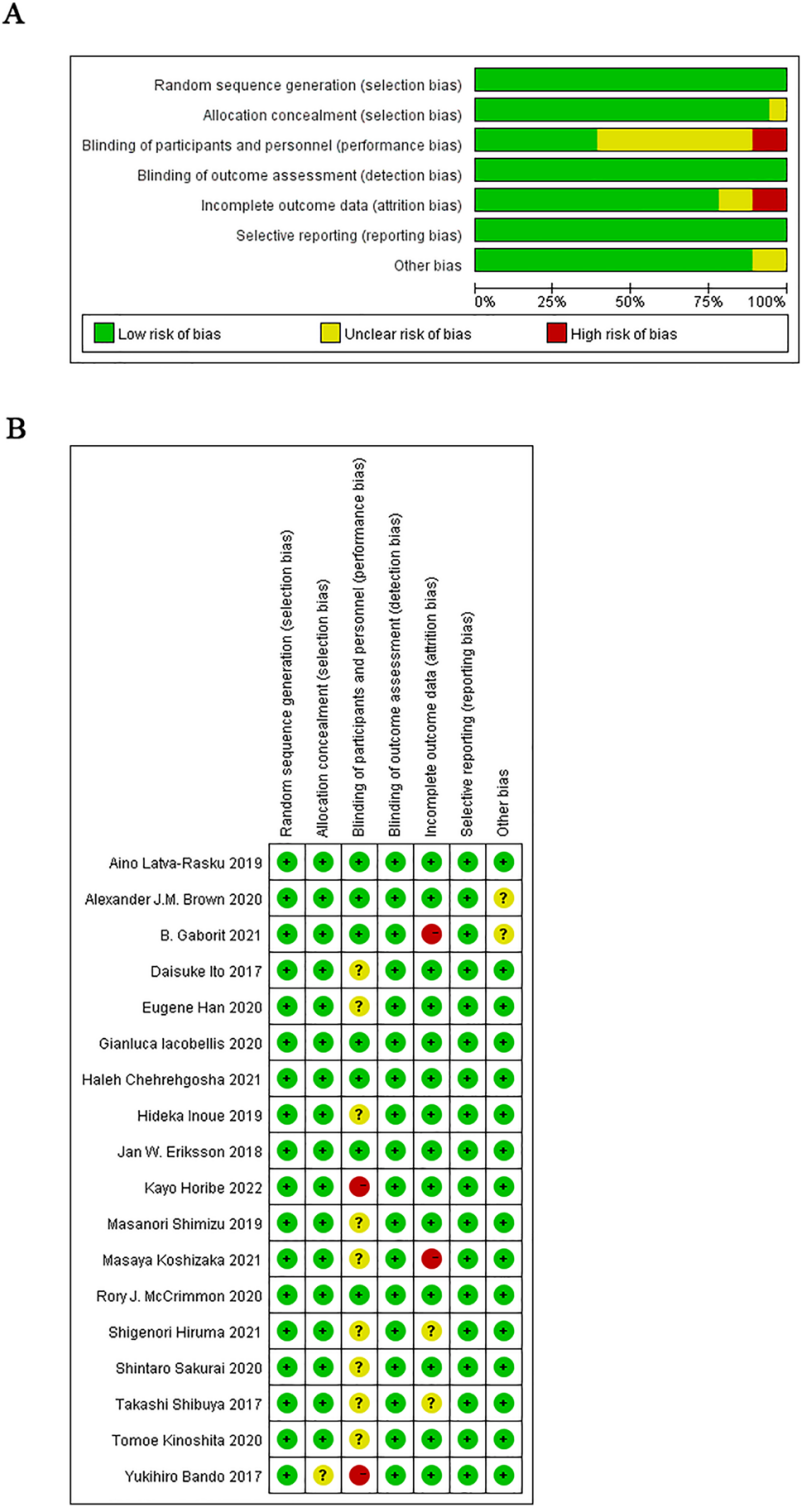
Methodological quality graph. **A** Methodological quality graph; **B** methodological quality summary

Figure 2 show the quality assessment of the included studies. In most trials, computer-generated random numbers were used for random assignment, and all trials evaluated the study results in a blinded manner. Because of the lack of blinding among the participants and examining personnel, many studies were rated as having medium risk. Other sources of bias included individual studies unclear methods on allocation concealment and reports with incomplete outcome data.

### Meta-analysis

#### Efficacy of SGLT-2 inhibitors on VAT reduction

**Fig. 3:**
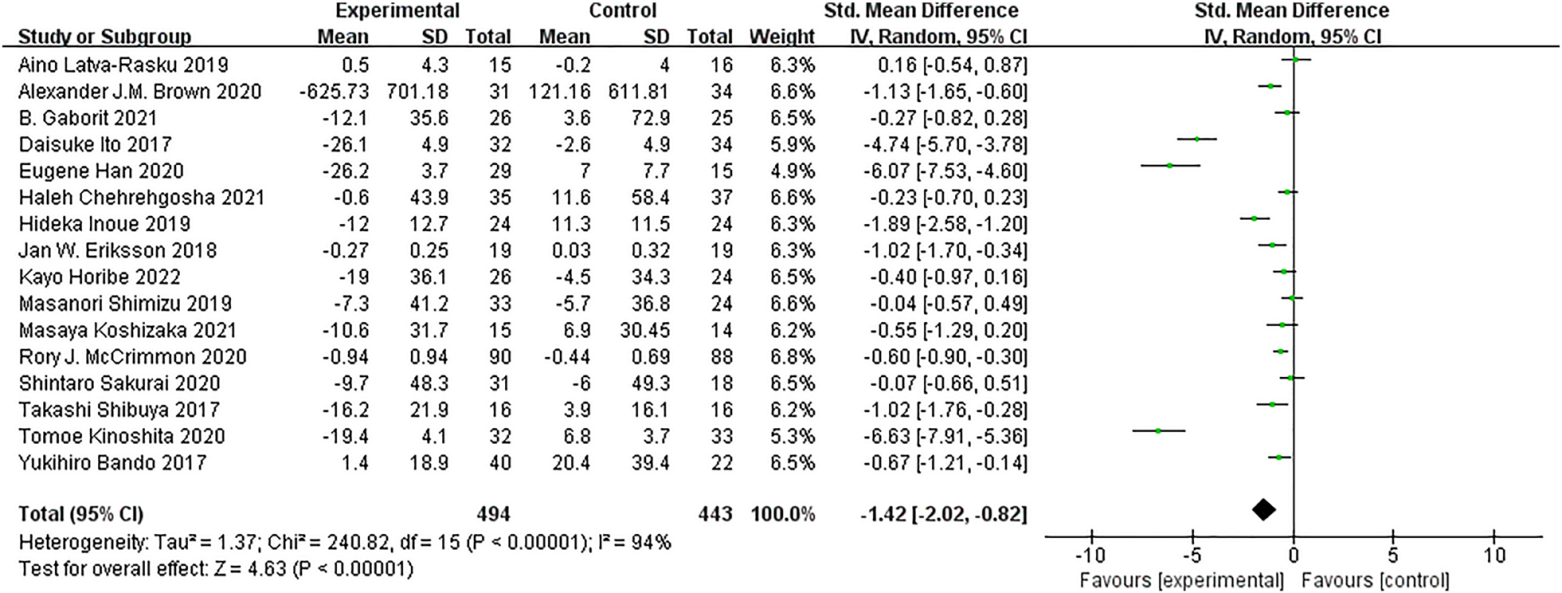
SGLT-2 inhibitors compared to control on VAT levels

A total of 16 RCTs involving 937 participants estimated VAT. Compared with the control group, VAT was significantly lower in the SGLT-2 inhibitor group (SMD = − 1.42; 95% CI [− 2.02, − 0.82]; I^2^ = 94%; *p* < 0.0001; Fig. 3). Owing to the high heterogeneity in the results, we performed subgroup analyses based on intervention duration, baseline BMI (obesity: BMI > 28 kg/m^2^; overweight: BMI 24–28 kg/m^2^), average age of patients, and whether or not to merge NAFLD with T2DM to analyse the sources of heterogeneity.

**Fig. 4:**
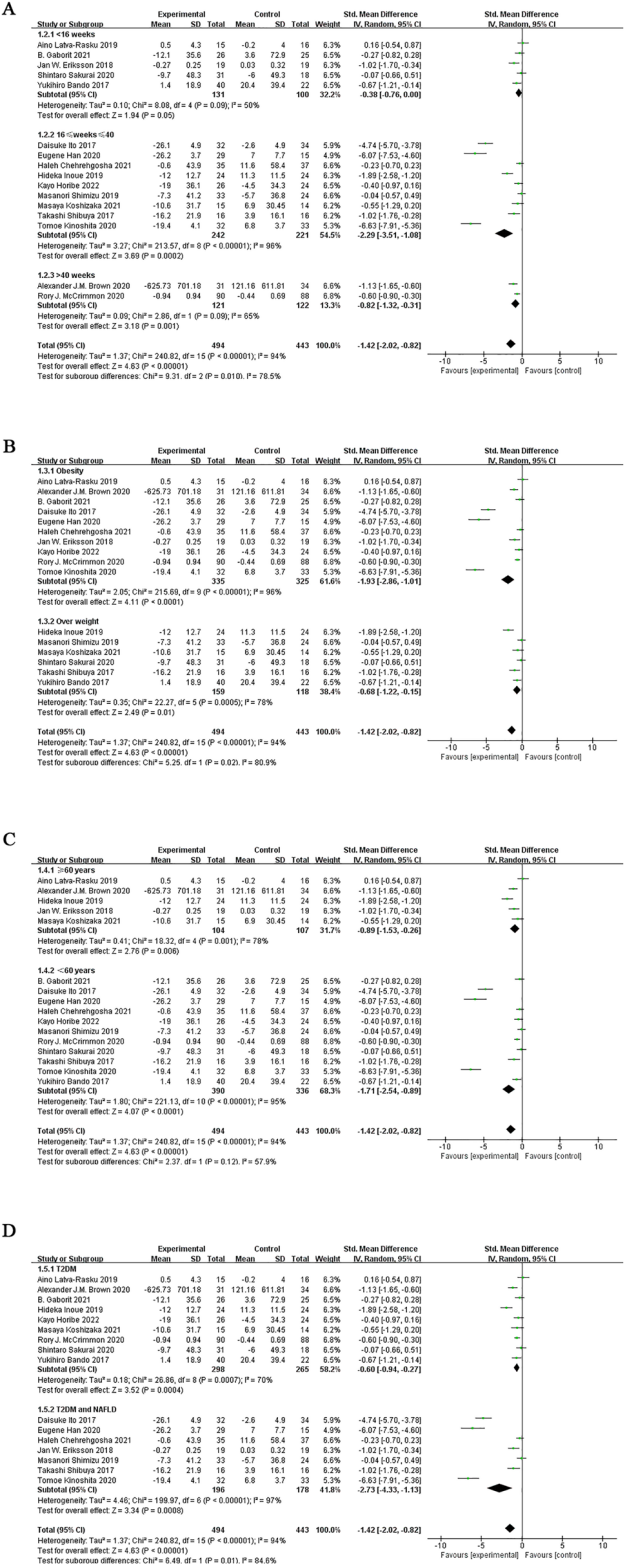
Subgroup analyses of SGLT-2 inhibitors efficacy on VAT. **A** Subgroup analysis of VAT based on the duration of the intervention; **B** subgroup analysis of VAT based on BMI; **C** subgroup analysis of VAT based on the age; **D** subgroup analysis of VAT based on T2DM with or without NAFLD

Subgroup analysis based on intervention duration showed that SGLT-2 inhibitors reduced VAT when they were administered between 16 to 40 weeks (SMD = − 2.29; 95% CI [− 3.51, − 1.08]; I^2^ = 96%; p = 0.0002) or more (SMD = − 0.82; 95% CI [− 1.32, − 0.31]; I^2^ = 65%; *p* = 0.001). However, when the administration time was 16 to 40 weeks, the SAT decreased more significantly. When they were administered with an intervention duration of < 16 weeks, they did not reduce VAT (SMD = − 0.38; 95% CI [− 0.76, 0.00]; I^2^ = 50%; *p* = 0.05; Fig. 4A).

Subgroup analyses based on baseline BMI showed that reduction in VAT due to the SGLT-2 inhibitor was significant for the obesity group (SMD = − 1.93; 95% CI [− 2.86, − 1.01]; I^2^ = 96%; *p* < 0.0001), as compared with the overweight group (SMD = − 0.68; 95% CI [− 1.22, − 0.15]; I^2^ = 78%; *p* = 0.01; Fig. 4B).

Subgroup analyses according to the mean age of patients included in the study showed that the SGLT-2 inhibitors significantly reduced VAT when the mean age was less than 60 years (SMD = − 1.71; 95% CI [− 2.54, − 0.89]; I^2^ = 95%; *p* < 0.0001) as compared with age more than 60 years (SMD = − 0.89; 95% CI [− 1.53, − 0.26]; I^2^ = 78%; *p* = 0.006; Fig. 4C).

Subgroup analysis based on whether or not NAFLD was merged with T2DM showed that the SGLT-2 inhibitor significantly reduced VAT in patients with NAFLD in T2DM (SMD = − 2.73; 95% CI [− 4.33, − 1.13]; I^2^ = 97%; *p* = 0.0008) compared with that in patients without NAFLD in T2DM (SMD = − 0.60; 95% CI [− 0.94, − 0.27]; I^2^ = 70%; *p* = 0.0004; Fig. 4D).

#### Efficacy of SGLT-2 inhibitors on SAT reduction

**Fig. 5:**
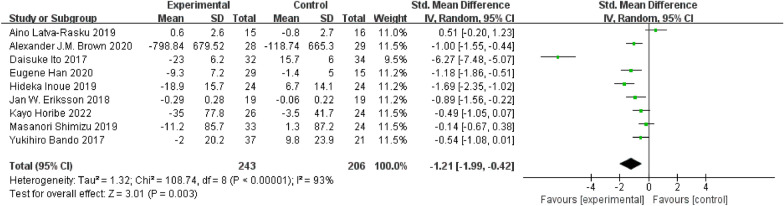
Effect of SGLT-2 inhibitors compared to control on SAT levels

A total of 16 RCTs involving 937 participants estimated VAT. Compared with the control group, SAT was significantly lower in the SGLT-2 inhibitor group (SMD = − 1.21; 95% CI [− 1.99, − 0.42]; I^2^ = 93%; *p* = 0.003; Fig. 5). Similarly, because of the high heterogeneity in the results, we conducted relevant subgroup analysis.

**Fig. 6:**
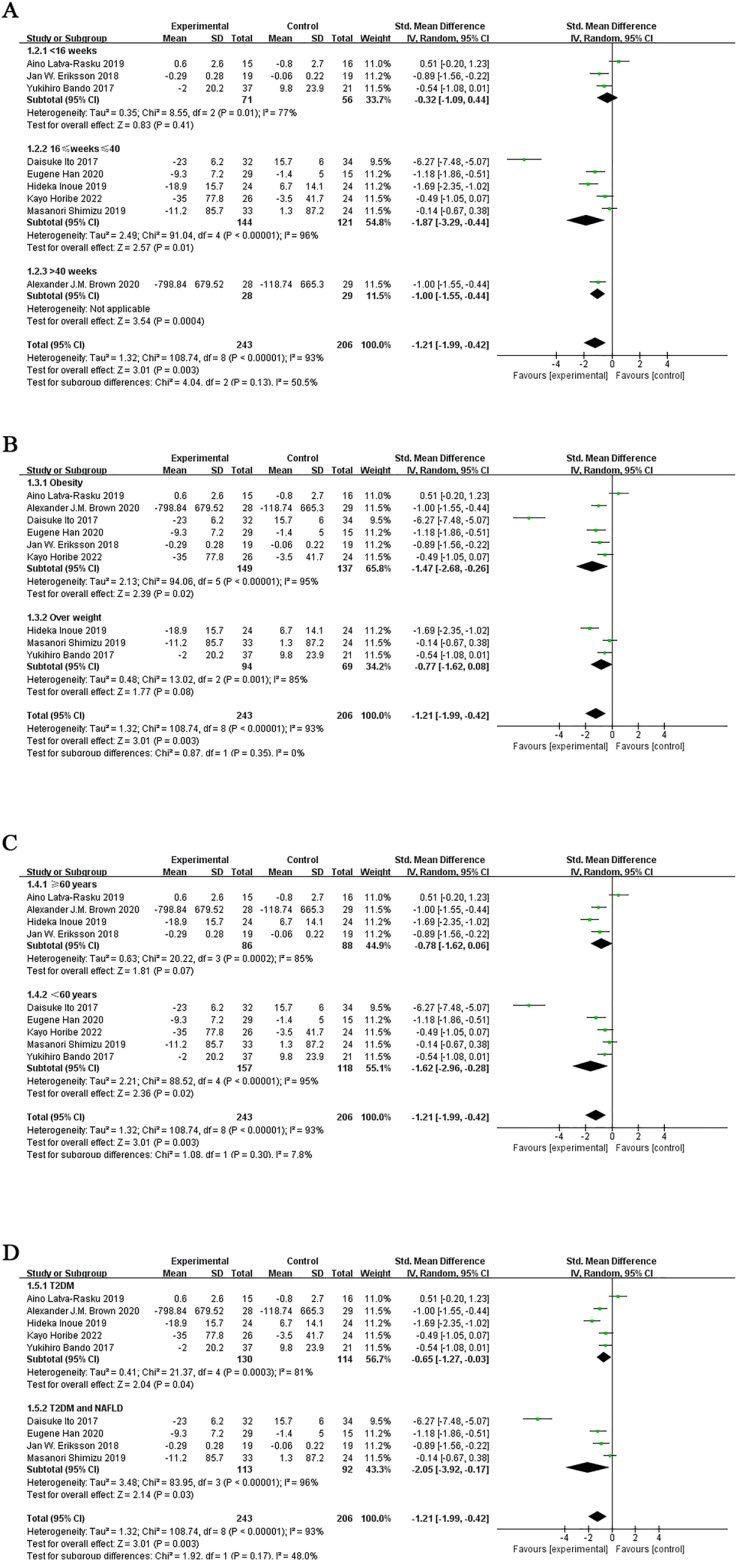
Subgroup analyses of SGLT-2 inhibitors efficacy on SAT. **A** Subgroup analysis of SAT based on the duration of the intervention; **B** subgroup analysis of SAT based on BMI; **C** subgroup analysis of SAT based on the age; **D** subgroup analysis of SAT based on T2DM with or without NAFLD

Subgroup analysis based on intervention duration showed that SGLT-2 inhibitors reduced SAT when they were administered between 16 and 40 weeks (SMD = − 1.87; 95% CI [− 3.29, − 0.44]; I^2^ = 96%; *p* = 0.01) or more (SMD = − 1.00; 95% CI [− 1.55, − 0.44]). However, when the administration time was 16 to 40 weeks, the SAT decreased more significantly. When the intervention duration was < 16 weeks (SMD = − 0.32; 95% CI [− 1.09, 0.44]; I^2^ = 77%; *p* = 0.41), SGLT-2 inhibitors did not reduce SAT (Fig. 6A).

Subgroup analyses based on baseline BMI showed that SGLT-2 inhibitors showed a significant reduction in SAT only in the obesity group (SMD = − 1.47; 95% CI [− 2.68, − 0.26]; I^2^ = 95%; *p* = 0.02; Fig. 6B).

Subgroup analyses according to mean patient age showed that the SGLT-2 inhibitors significantly reduced SAT only when the mean age was below 60 years (SMD = − 1.62; 95% CI [− 2.96, − 0.28]; I^2^ = 95%; *p* = 0.02; Fig. 6C).

Subgroup analysis based on whether or not NAFLD was merged with T2DM showed that SGLT-2 inhibitors significantly reduced SAT in patients with NAFLD in T2DM (SMD = − 2.05; 95% CI [− 3.92, − 0.17]; I^2^ = 96%; *p* = 0.03) compared with that in patients without NAFLD in T2DM (SMD = − 0.65; 95% CI [− 1.27, − 0.03]; I^2^ = 81%; *p* = 0.04; Fig. 6D).

#### Efficacy of SGLT-2 inhibitors on ectopic fat

**Fig. 7:**
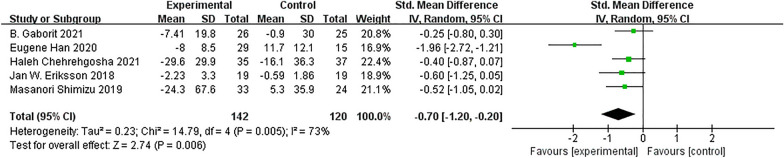
Effect of SGLT-2 inhibitors compared to control on liver fat levels

Five studies involving 262 participants evaluated the effects of SGLT-2 inhibitors on liver fat and compared with placebo or other antihyperglycemic drugs; the results showed that SGLT-2 inhibitors significantly decreased liver fat (SMD = − 0.70; 95% CI [− 1.20, − 0.20]; I^2^ = 73%; *p* = 0.006; Fig. 7).

Subgroup analysis based on intervention duration showed that SGLT-2 inhibitors reduced liver fat only when they were administered between 16 and 40 weeks (SMD = − 0.91; 95% CI [− 1.75, − 0.07]; I^2^ = 84%; *p* = 0.03) as compared with < 16 weeks (SMD = − 0.40; 95% CI [− 0.82, 0.02]; I^2^ = 0%; *p* = 0.06; Additional file 1: Fig. S1A and Table S1).

Subgroup analyses based on baseline BMI showed that reduction in liver fat due to the SGLT-2 inhibitor was significant for only on the obesity group (SMD = − 0.76; 95% CI [− 1.42, − 0.10]; I^2^ = 80%; *p* = 0.02), as compared with the overweight group (SMD = − 0.52; 95% CI [− 1.05, 0.02]; *p* = 0.06; Additional file 1: Fig. S1B and Table S1).

Subgroup analyses according to mean patient age showed that the SGLT-2 inhibitors significantly reduced liver fat only when the mean age was below 60 years (SMD = − 0.73; 95% CI [− 1.36, − 0.11]; I^2^ = 80%; *p* = 0.02; Additional file 1: Fig. S1C and Table S1).

Subgroup analysis based on whether or not NAFLD was merged with T2DM showed that SGLT-2 inhibitors significantly reduced liver fat only in patients with NAFLD in T2DM (SMD = − 0.82; 95% CI [− 1.43, − 0.21]; I^2^ = 77%; *p* = 0.008) compared with that in patients without NAFLD in T2DM (SMD = − 0.25; 95% CI [− 0.80, 0.30]; *p* = 0.37; Additional file 1: Fig. S1D and Table S1).

**Fig. 8:**
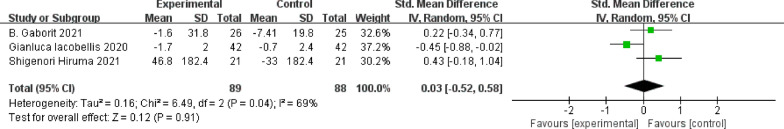
Effect of SGLT-2 inhibitors compared to control on EAT levels

Only 3 trials reported EAT, and the results were compared between the treatment group and the control group; SGLT-2 inhibitors did not reduce EAT (SMD = 0.03; 95% CI [− 0.52, 0.58]; I^2^ = 69%; *p* = 0.91; Fig. 8).

#### Changes in other body composition

**Fig. 9:**
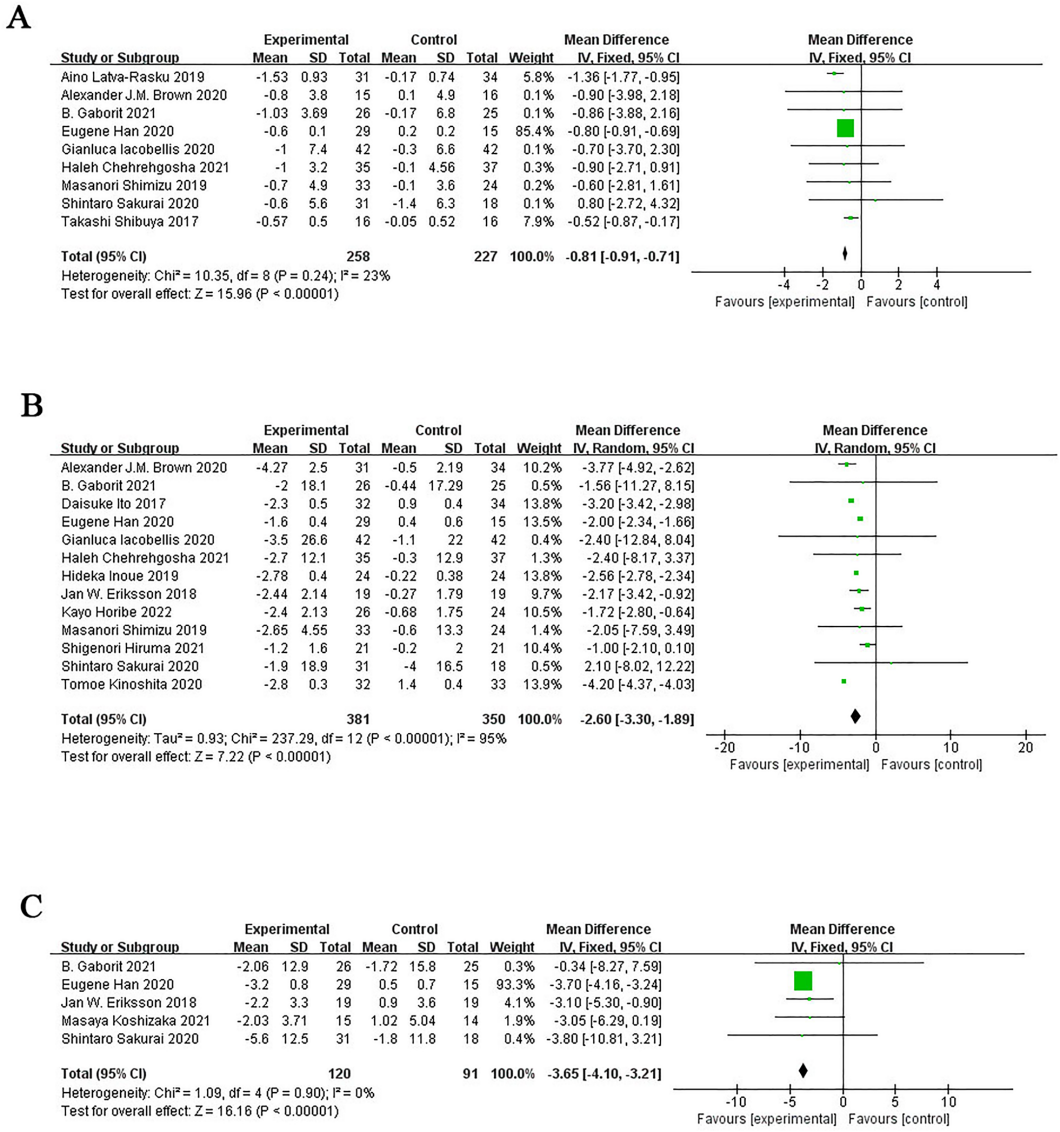
Effect of SGLT-2 inhibitors compared to control on BMI levels (**A**); effect of SGLT-2 inhibitors compared to control on body weight levels (**B**); effect of SGLT-2 inhibitors compared to control on waist circumference levels (**C**)

The results of anthropometric parameters in the SGLT-2 inhibitor group showed a significant reduction. Compared with the control group, the SGLT-2 inhibitor group significantly reduced BMI (MD = − 0.81; 95% CI [− 0.91, − 0.71]; I^2^ = 23%; *p* < 0.0001), body weight (MD = − 2.60; 95% CI [− 3.30, − 1.89]; I^2^ = 95%; *p* < 0.0001), and waist circumference (MD = − 3.65; 95% CI [− 4.10, − 3.21]; I^2^ = 0%; *p* < 0.0001; Fig. 9). Due to the low heterogeneity of BMI and waist circumference, sensitivity analysis and subgroup analysis were not required to determine the source of heterogeneity. Only a subgroup analysis of body weight levels was performed to determine the source of heterogeneity.

Subgroup analysis based on intervention duration showed that SGLT-2 inhibitors reduced body weight when they were administered between 16 and 40 weeks (SMD = − 2.77; 95% CI [− 3.61, − 1.93]; I^2^ = 97%; *p* < 0.0001) or more (SMD = −3.77; 95% CI [− 4.92, − 2.62]; *p* < 0.0001) (Additional file 1: Fig. S2A and Table S2).

Subgroup analyses based on baseline BMI showed that reduction in body weight due to the SGLT-2 inhibitor was significant for the overweight group (SMD = − 2.56; 95% CI [− 2.78, − 2.34]; I^2^ = 0; *p* < 0.0001), and obesity group (SMD = − 2.63; 95% CI [− 3.45, − 1.81]; I^2^ = 95%; *p* < 0.0001; Additional file 1: Fig. S2B and Table S2).

Subgroup analyses according to the mean age of patients included in the study showed that the SGLT-2 inhibitors significantly reduced body weight when the mean age was less than 60 years (SMD = − 2.46; 95% CI [− 3.40, − 1.53]; I^2^ = 95%; *p* < 0.0001) and age more than 60 years (SMD = − 2.77; 95% CI [− 3.51, − 2.03]; I^2^ = 56%; *p* < 0.0001; Additional file 1: Fig. S2C and Table S2).

Subgroup analysis based on whether or not NAFLD was merged with T2DM showed that SGLT-2 inhibitors significantly reduced body weight in patients with NAFLD in T2DM (SMD = − 2.91; 95% CI [− 3.91, − 1.92]; I^2^ = 97%; *p* < 0.0001) compared with that in patients without NAFLD in T2DM (SMD = − 2.25; 95% CI [− 3.08, − 1.43]; I^2^ = 60%; *p* < 0.0001; Additional file 1: Fig. S2D and Table S2).

#### Sensitivity analysis and publication bias

**Fig. 10:**
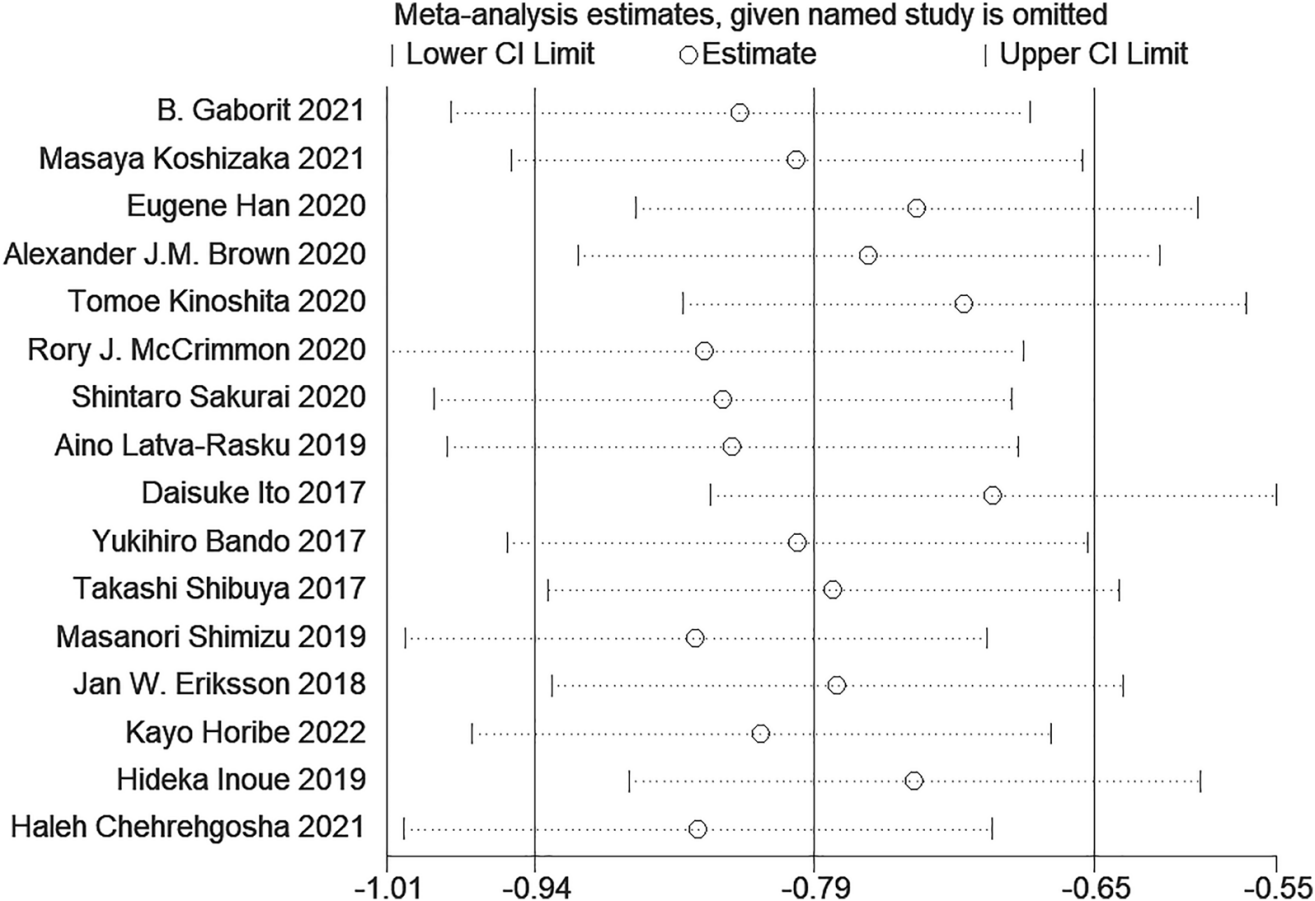
Sensitivity analysis of VAT

**Fig. 11:**
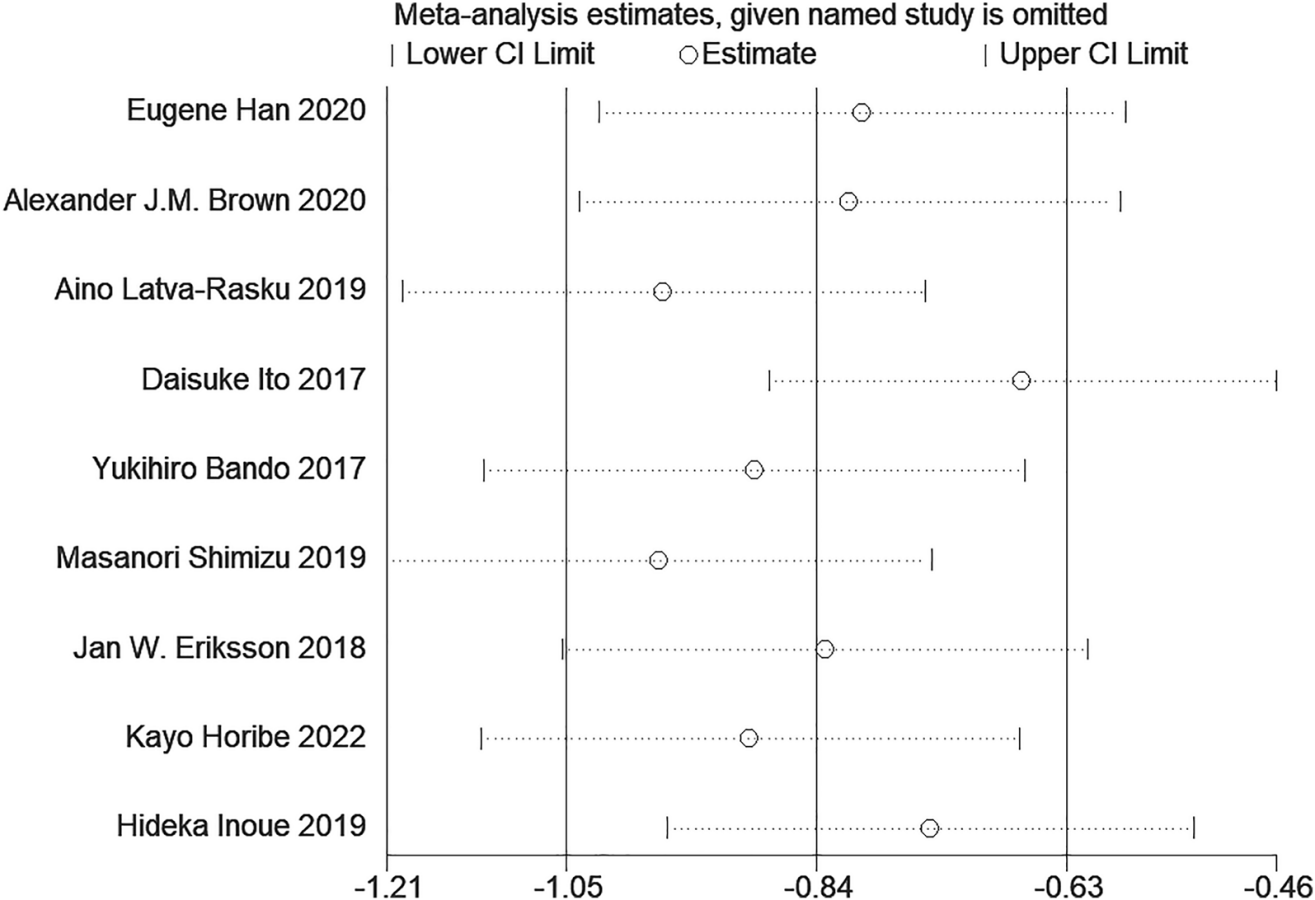
Sensitivity analysis of SAT

**Fig. 12:**
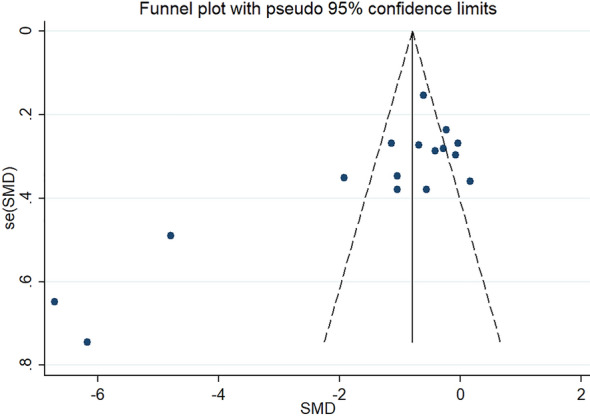
Funnel plot of VAT

**Fig. 13:**
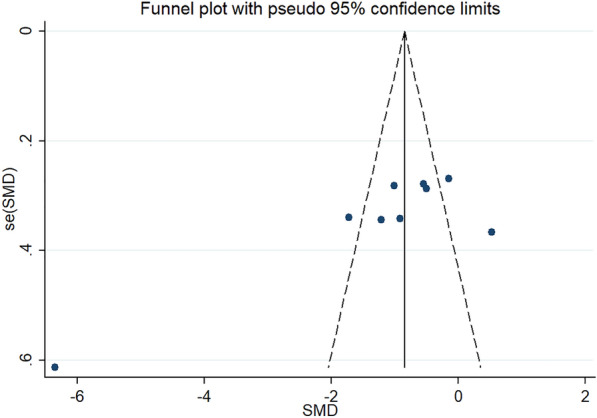
Funnel plot of SAT

Because of the high heterogeneity in the research results for SAT, VAT, liver fat and body weight, we explored the sources of heterogeneity through sensitivity analysis. The sensitivity analysis results of VAT, SAT, liver fat and body weight showed that any single study excluded from the study would not affect the significance of our combined effect size on any result (Figs. 10, 11, Additional file 1: Figs. S5, S6). The funnel diagram of VAT showed asymmetry (Fig. 12). We further conducted Begg’s test (*p* = 0.251), and the results indicated that no publication bias was evident. The funnel diagram of SAT showed symmetry (Fig. 13), indicating that the included articles had no publication bias and the results were robust.

## Discussion

This meta-analysis included 18 RCTs involving 1063 participants. The results of the meta-analysis showed that, compared with other antidiabetic drugs or placebo, SGLT-2 inhibitors reduced VAT, SAT, and ectopic liver fat tissue and exerted significant effects on reducing body weight, waist circumference, and BMI.

Apart from their well-established hypoglycemic effects, SGLT-2 inhibitors have also demonstrated the ability to mitigate long-term cardiovascular complications. Previous research [9] has indicated that SGLT-2 inhibitors significantly reduce the risk of cardiovascular events and all-cause mortality in type 2 diabetes patients with high cardiovascular risk. Given that VAT, SAT and ectopic fat are important cardiovascular risk factors [5, 6, 37] the researchers propose that the cardiac metabolic effects of SGLT-2 inhibitors might be mediated through the reduction of VAT, SAT and ectopic fat. More importantly, subgroup analysis showed that SGLT-2 inhibitors are more effective in reducing VAT, SAT and ecpotic liver fat in patients with T2DM combined with NAFLD, but due to significant heterogeneity, we should treat this result with caution. The risk of coronary heart disease was significantly higher in patients with NAFLD than in the general population, which may be caused by inflammation and dysfunctional adipose tissue [38]. The E-LIFT trial showed [39] that SGLT-2 inhibitors can improve hepatic steatosis and fibrosis T2DM patients with NAFLD and reduce alanine aminotransferase (ALT) levels. A recent meta-analysis by Wei et al. showed that [40] SGLT-2 inhibitors effectively improved NAFLD by reducing liver enzymes and liver fat and improving body composition. Xing et al. also conducted a meta-analysis of six RCTs [41], involving 309 patients with NAFLD and T2DM and concluded that SGLT-2 inhibitors significantly reduced ALT and liver fat content, consistent with our research results, confirming the reliability of our research results. Because T2DM and NAFLD are bidirectional, improvement in NAFLD reduces the cardiovascular risk of T2DM [42]. Considering the associations between the accumulation of adipose tissue (VAT, SAT and ectopic fat) and NAFLD with an increased risk of cardiometabolic disorders [6, 37, 42]. Therapeutic interventions targeting the reduction of NAFLD and excessive VAT, SAT and ectopic fat could potentially yield favorable outcomes for cardiac metabolic health. Therefore, the use of SGLT-2 inhibitors is more applicable to patients with T2DM at high risk of cardiac metabolic disease, rather than restricting its use for weight loss and hypoglycemic effect in patients with T2DM.

In addition, subgroup analysis showed that when SGLT2 was used for less than 16 weeks, there was no significant difference in fat reduction. This result shows that in order to achieve the cardiovascular benefits of SGLT2 inhibitor mediated by visceral fat reduction, it needs more than 16 weeks of administration; However, treatment for more than 40 weeks does not guarantee better efficacy. Prolonged duration may lead to resistance to SGLT2 inhibitors. It is suggested that appropriate administration time (16–40 weeks) may provide more significant and stable beneficial effects in reducing VAT and SAT.

Interestingly, our study found that SGLT-2 inhibitors could significantly reduce VAT, SAT and ectopic fat in T2DM patients with higher baseline BMI at a young age. In a meta-analysis [43] of 55 RCTs, treatment with SGLT-2 inhibitors was significantly associated with weight loss in patients with T2DM when compared with that with placebo, and a dose-dependent response in body weight reduction to dapagliflozin was observed. It is reported that if the weight loss is 3%, it can significantly reduce the cardiovascular risk of obese T2DM patients [44]. Weight loss caused by SGLT-2 inhibitor treatment plays an important role in reducing visceral fat and thus cardiovascular risk. The evidence provided by our research shows that there is a strong correlation between the reduction of adipose tissue and BMI, especially in patients with higher BMI, the reduction of VAT, SAT and ectopic fat is more significant. Considering the potential advantages of reducing VAT, SAT and ectopic fat for cardiac metabolism, SGLT-2 inhibitors can be considered for primary prevention in obese patients with TD2M. In the future, we may be able to use VAT, SAT and ectopic fat content and its reduction to evaluate the preventive and therapeutic efficacy of SGLT-2 inhibitors in obese patients with TD2M.

Excessive accumulation of VAT and ectopic adipose is closely related to insulin resistance, metabolic syndrome, and atherosclerosis [45]. However, reduction of VAT and ectopic adipose can reduce the risk of cardiovascular disease in patients with T2DM and decrease the impact of metabolic disorders. Although the positive effects of lifestyle modification and weight loss surgery on VAT and ectopic adipose tissue have been previously confirmed [46, 47], there are no specially developed drugs to reduce VAT and ectopic adipose tissue. SGLT-2 inhibitors exert cardioprotective effects independent of hypoglycemia [9]. In clinical studies, treatment with SGLT-2 inhibitors can improve insulin sensitivity [48] and reduce body weight [49]. Recently, in a 24-week RCT study [33], body weight and visceral fat area of patients with T2DM and NAFLD were compared between the ipragliflozin and pioglitazone groups, and only the ipragliflozin group had a significant reduction in the two parameters. It is speculated that SGLT-2 inhibitors, as a hypoglycemic drug, may be a therapeutic approach for reducing the adipose tissue.

SGLT-2 inhibitors can target the EAT of left atrial and coronary artery to treat and prevent atrial fibrillation and coronary artery disease by reducing visceral fat inflammation and increasing free fatty acid oxidation [50]. However, our study found that SGLT-2 inhibitors do not reduce EAT. Because only fewer studies have assessed EAT, large-scale clinical studies are needed to evaluate the effects of SGLT-2 inhibitors on EAT content.

However, the mechanism by which SGLT-2 inhibitors reduce the adipose tissue remains unclear. Previous experimental and clinical studies have suggested some potential mechanisms. On the one hand, SGLT-2 inhibitors can improve insulin resistance, increase glucose excretion in urine by competitively blocking sodium–glucose co-transporter in the renal proximal convoluted tubules, reduce insulin level, improve insulin resistance and reduce stimulation of fat regeneration [51]. The improvement of insulin resistance leads to the downregulation of sterol regulatory element–binding protein 1c (SREBP‐1c), which is a transcription factor involved in the activation of de novo adipogenesis [52]. On the other hand, glucagon plays an important role. SGLT-2 in present in islet α cells (expressed by the SLC5A2 gene), and inhibition of this sodium–glucose co-transporter results in increased glucagon secretion [53]. Glucagon activates PPAR-α [54] and CPT-1A [55] and stimulates the increase in gluconeogenesis and liver fat β-oxidation, leading to the conversion of carbohydrate metabolism to fatty acid metabolism, which helps reduce liver fat deposition [56]. An in vivo study reported that the SGLT-2 inhibitor canagliflozin can activate AMPK in the liver of mice, thus inhibiting respiratory chain complex I and consequently lipid synthesis [57].

This meta-analysis has several limitations. First, the results of this study have high heterogeneity, and therefore, we need to interpret the results with caution. Despite subgroup and sensitivity analyses failing to identify the source of heterogeneity, upon meticulous examination of the included studies, we speculate that the heterogeneity could be due to differences in the inclusion and exclusion criteria, such as the fluctuations in the HbA1c and BMI ranges in the included population. Furthermore, the primary and key secondary endpoints of the included studies were different, and the measures evaluated in this study were not the primary objectives of most trials, which could have also contributed to the observed heterogeneity. In the future, this can be further verified by designing large-scale clinical studies with adipose tissue assessment as the endpoint. Additionally, the included studies used different measurement methods to assess the measured indicators, which led to inevitable measurement errors. Although subgroup analysis based on different measurement methods showed a slight decrease in heterogeneity (Additional file 1: Figs. S3, S4), the differences in experience and methods of the measuring personnel may have resulted in some bias in the collected data. Finally, it should be noted that the published evidence is limited, and the studies we included, while conducted in multiple countries, were primarily based on small randomized clinical trials. Therefore, conducting large-scale randomized controlled trials in the future will be necessary to confirm these findings.

## Conclusions

In T2DM patients, SGLT-2 inhibitors significantly reduced the VAT, SAT and ectopic liver fat of patients, especially in young T2DM patients with NAFLD and high BMI, and treatment for more than 40 weeks does not guarantee better efficacy, appropriate administration time (16–40 weeks) may provide more significant and stable beneficial effects in reducing VAT and SAT. Our results may support that SGLT-2 is more suitable for obese or NAFLD patients with high risk of cardiovascular metabolic disease in type 2 diabetes.

## Supplementary Information

**Additional file 1: Text S1.** Comprehensive search strategy. **Figure S1.** Subgroup analysis of SGLT-2 inhibitors efficacy on liver fat. **Figure S2.** Subgroup analysis of SGLT-2 inhibitors efficacy on body weight. **Figure S3.** Subgroup analysis of SGLT-2 inhibitors efficacy on VAT. **Figure S4.** Subgroup analysis of SGLT-2 inhibitors efficacy on SAT. **Table S1.** Subgroup analysis of SGLT-2 inhibitors efficacy on liver fat. **Table S2.** Subgroup analysis of SGLT-2 inhibitors efficacy on body weight. **Figure S5.** Sensitivity analysis of liver fat levels. **Figure S6.** Sensitivity analysis of body weight level.

## Data Availability

The datasets generated and/or analyzed during the current study are available from the corresponding author on reasonable request.
