# Supplementary material for: Effects of SGLT-2 inhibitors on adipose tissue distribution in patients with type 2 diabetes mellitus: a systematic review and meta-analysis of randomized controlled trials
Source: Diabetol Metab Syndr. 2023 May 31;15:113. doi: 10.1186/s13098-023-01085-y (PMC10230759; doi:10.1186/s13098-023-01085-y)
Supplement: Supplementary file 1 — Additional file 1: Text S1. Comprehensive search strategy. Figure S1. Subgroup analysis of SGLT-2 inhibitors efficacy on liver fat. Figure S2. Subgroup analysis of SGLT-2 inhibitors efficacy on body weight. Figure S3. Subgroup analysis of SGLT-2 inhibitors efficacy on VAT. Figure S4. Subgroup analysis of SGLT-2 inhibitors efficacy on SAT. Table S1. Subgroup analysis of SGLT-2 inhibitors efficacy on liver fat. Table S2. Subgroup analysis of SGLT-2 inhibitors efficacy on body weight. Figure S5. Sensitivity analysis of liver fat levels. Figure S6. Sensitivity analysis of body weight level. [file 13098_2023_1085_MOESM1_ESM.docx]

**Additional file 1**

**pubmed Search Strategy**

1. (((((((((((((((((((((((("Sodium-Glucose Transporter 2 Inhibitors"[Mesh]) OR (Sodium Glucose Transporter 2 Inhibitors)) OR (SGLT-2 Inhibitors)) OR (SGLT 2 Inhibitors)) OR (SGLT2 Inhibitors)) OR (Sodium-Glucose Transporter 2 Inhibitor)) OR (Sodium Glucose Transporter 2 Inhibitor)) OR (SGLT2 Inhibitor)) OR (Inhibitor, SGLT2)) OR (Gliflozins)) OR (Gliflozin)) OR (SGLT-2 Inhibitor)) OR (Inhibitor, SGLT-2)) OR (SGLT 2 Inhibitor)) OR (Sodium-Glucose Transporter 2 Inhibitors)) OR (dapagliflozin)) OR (empagliflozin)) OR (canagliflozin)) OR (tofogliflozin)) OR (luseogliflozin)) OR (ertugliflozin)) OR (ipragliflozin)) OR (remogliflozin)) OR (sergliflozin))
2. (((((((adipose tissue) OR (adipose fat)) OR (subcutaneous fat)) OR (subcutaneous adipose)) OR (visceral fat)) OR (visceral adipose)) OR (ectopic fat)) OR (ectopic adipose)
3. (("Randomized Controlled Trial" [Publication Type]) OR (randomized)) OR (placebo)
4. #1 and #2 and #3

**Cochrane Search Strategy**

1. MeSH descriptor:[Sodium-Glucose Transporter 2 Inhibitors] explode all trees
2. (SGLT 2 Inhibitor) or (Sodium Glucose Transporter 2 Inhibitor) or (Sodium-Glucose Transporter 2 Inhibitor) or (SGLT-2 Inhibitor) or (SGLT 2 Inhibitors) or (Gliflozin) or (SGLT2 Inhibitor or (SGLT-2 Inhibitors) or (Sodium Glucose Transporter 2 Inhibitors) or (Inhibitor, SGLT-2) or (Inhibitor, SGLT2) or (SGLT2 Inhibitors or Gliflozins) or (dapagliflozin) or (empagliflozin or (canagliflozin) or (tofogliflozin) or (luseogliflozin) or (ertugliflozin or (ipragliflozin) or (remogliflozin) or (sergliflozin)
3. (adipose tissue) or (adipose fat) or (visceral fat) or (visceral adipose) or (ectopic fat)or (ectopic adipose) or (subcutaneous fat) or (subcutaneous fat)
4. (randomized Controlled Trial) or (randomized) or (placebo)
5. #2 and #3 and #4

**Embase Search Strategy**

#1 ‘SGLT2 Inhibitor’ or ‘Sodium Glucose Transporter 2 Inhibitor’ or ‘Sodium-Glucose Transporter 2 Inhibitor’ or ‘SGLT-2 Inhibitor’ or ‘SGLT 2 Inhibitors’ or ‘Gliflozin’ or ‘SGLT2 Inhibitor’ or ‘SGLT-2 Inhibitors’ or ‘Sodium Glucose Transporter 2 Inhibitors’ or ‘Inhibitor, SGLT-2’ or ‘Inhibitor, SGLT2’ or ‘SGLT2 Inhibitors’ or ‘Gliflozins’ or ‘dapagliflozin’ or ‘empagliflozin’ or ‘canagliflozin’ or ‘tofogliflozin’ or ‘luseogliflozin’ or ‘ertugliflozin’ or ‘ipragliflozin’ or ‘remogliflozin’ or ‘sergliflozin’

#2 ‘adipose tissue’ or ‘adipose fat’ or ‘visceral fat’ or ‘visceral adipose’ or ‘visceral fat’ or ‘visceral adipose’ or ‘ectopic fat’ or ‘ectopic adipose’

#3‘randomized Controlled Trial’ or ‘randomized’ or ‘placebo’

#4 #1 AND #2 AND #3

**ClinicalTrials.gov Search Strategy**

(Sodium Glucose Transporter 2 Inhibitors OR SGLT-2 Inhibitors OR SGLT 2 Inhibitors OR SGLT2 Inhibitors OR Sodium-Glucose Transporter 2 Inhibitor OR Sodium Glucose Transporter 2 Inhibitor OR SGLT2 Inhibitor OR Inhibitor, SGLT2 OR Gliflozins OR Gliflozin OR SGLT-2 Inhibitor OR Inhibitor, SGLT-2 OR SGLT 2 Inhibitor OR Sodium-Glucose Transporter 2 Inhibitors OR dapagliflozin OR empagliflozin OR canagliflozin OR tofogliflozin OR luseogliflozin OR ertugliflozin OR ipragliflozin OR remogliflozin OR sergliflozin) AND (adipose tissue OR adipose fat OR subcutaneous fat OR subcutaneous adipose OR visceral fat OR visceral adipose OR ectopic fat OR ectopic adipose) AND (placebo)


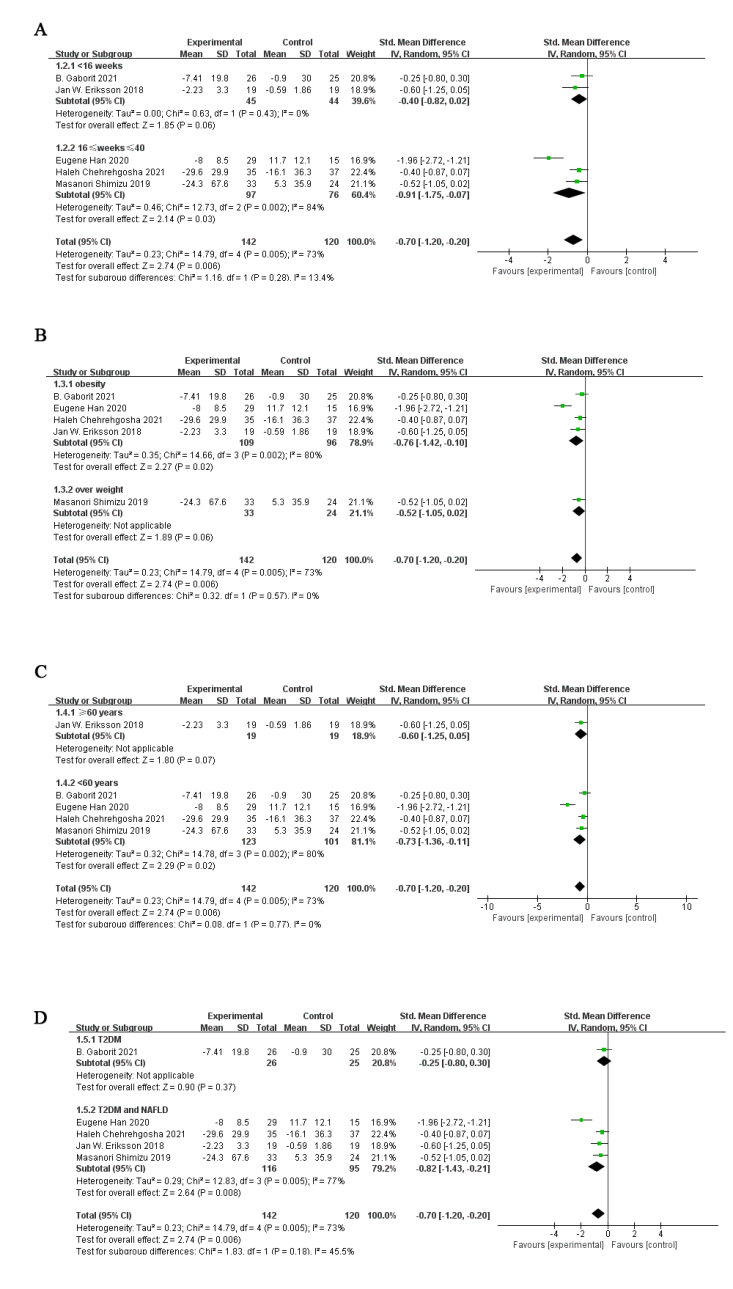


FIGURE S1 Subgroup analysis of SGLT-2 inhibitors efficacy on liver fat. (A) Subgroup analysis of liver fat based on the duration of the intervention ; (B) Subgroup analysis of liver fat based on BMI ; (C) Subgroup analysis of liver fat based on the age; (D) Subgroup analysis of liver fat based on T2DM with or without NAFLD.


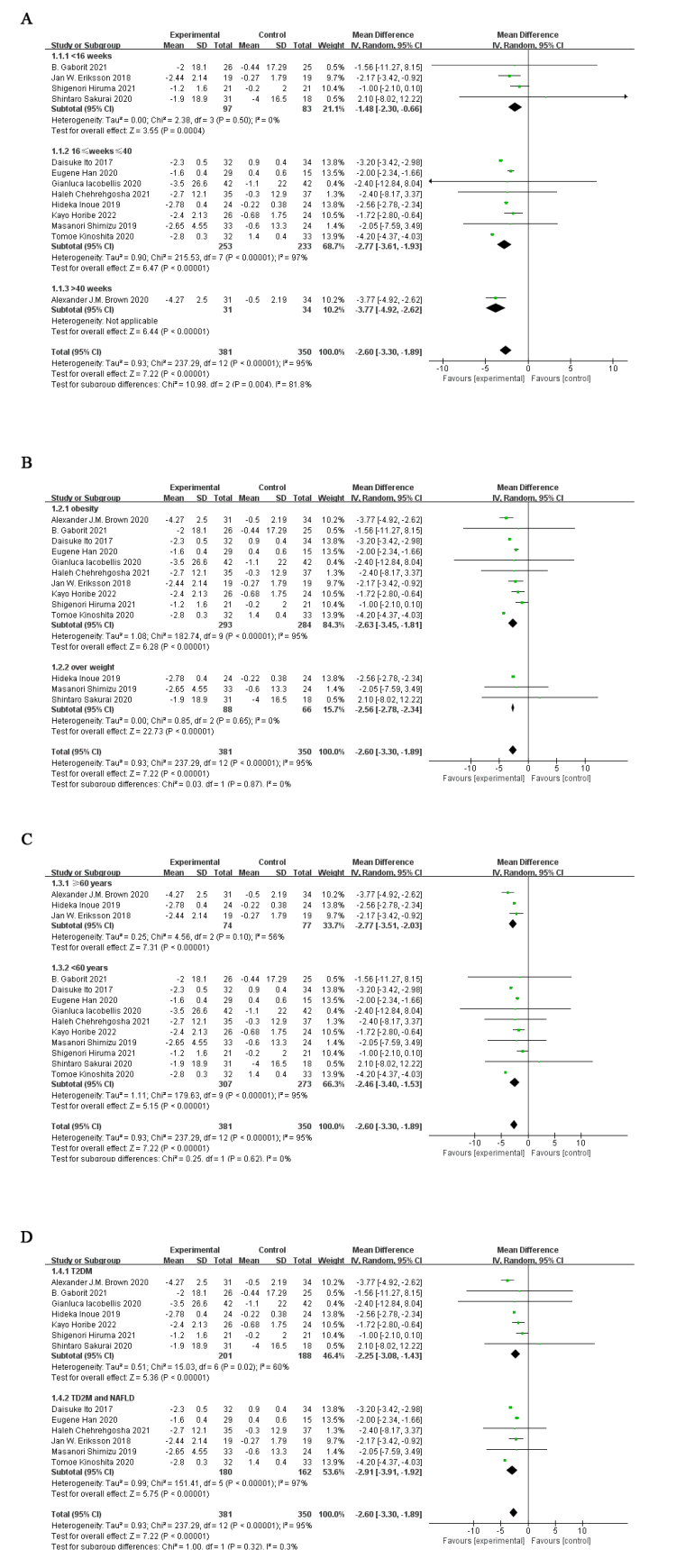


FIGURE S2 Subgroup analysis of SGLT-2 inhibitors efficacy on body weight. (A) Subgroup analysis of body weight based on the duration of the intervention ; (B) Subgroup analysis of body weight based on BMI ; (C) Subgroup analysis of body weight based on the age; (D) Subgroup analysis of body weight based on T2DM with or without NAFLD.


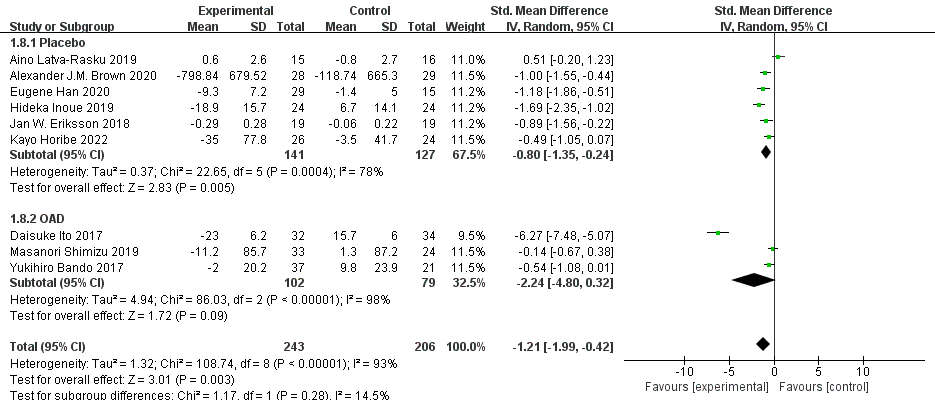

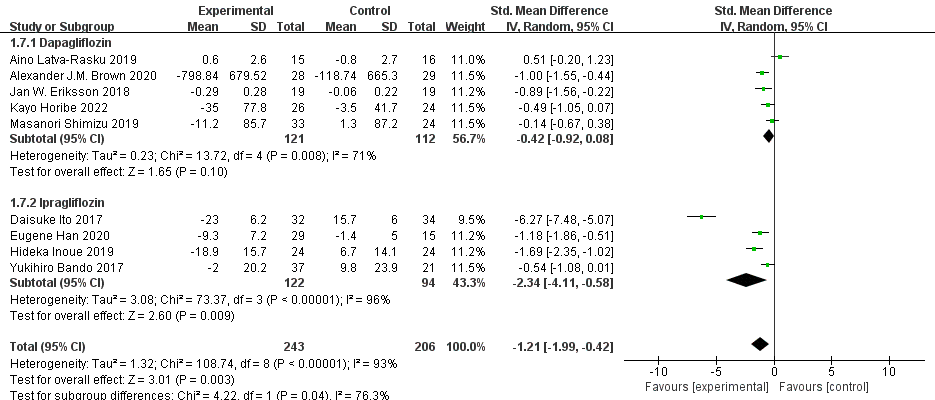

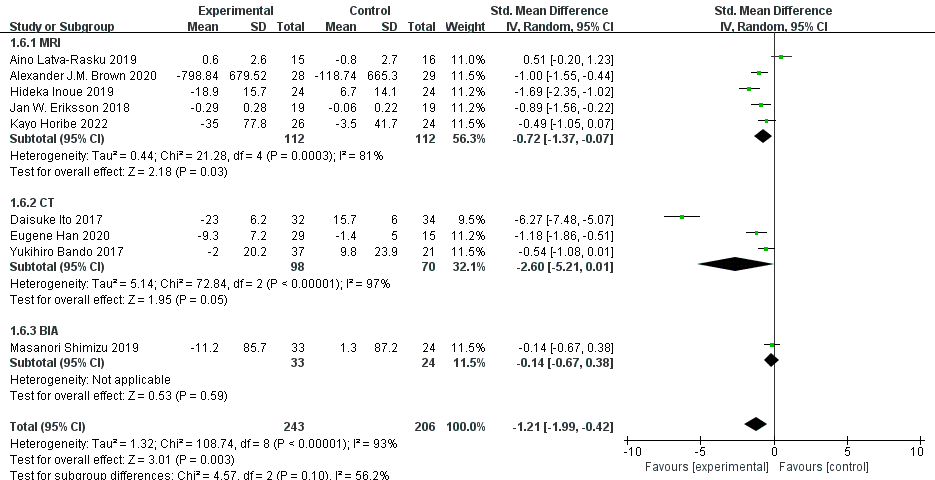

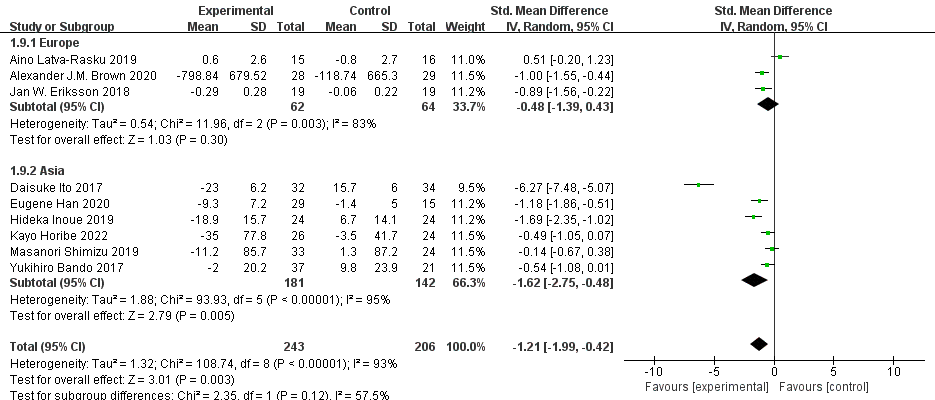


**A**

**B**

**C**

**D**


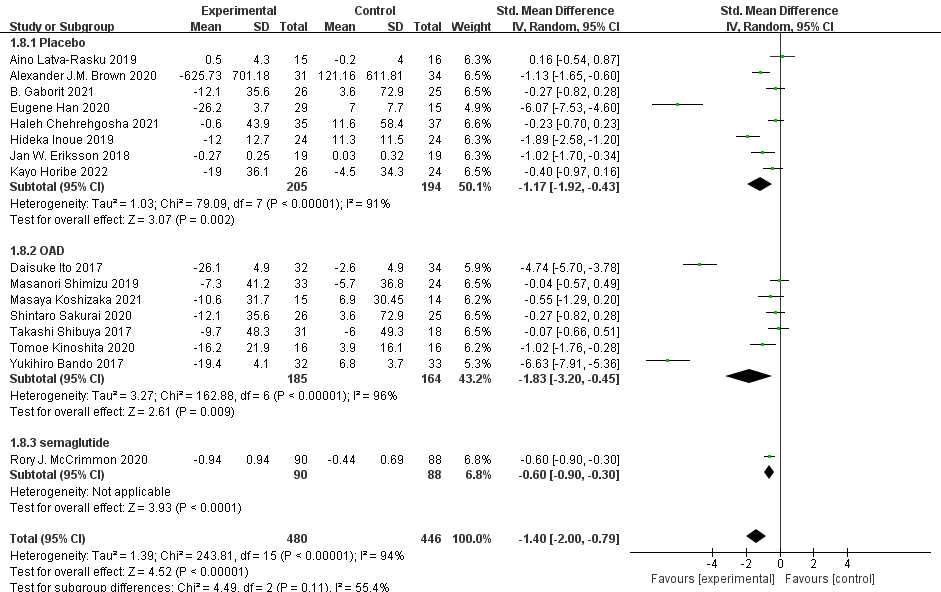

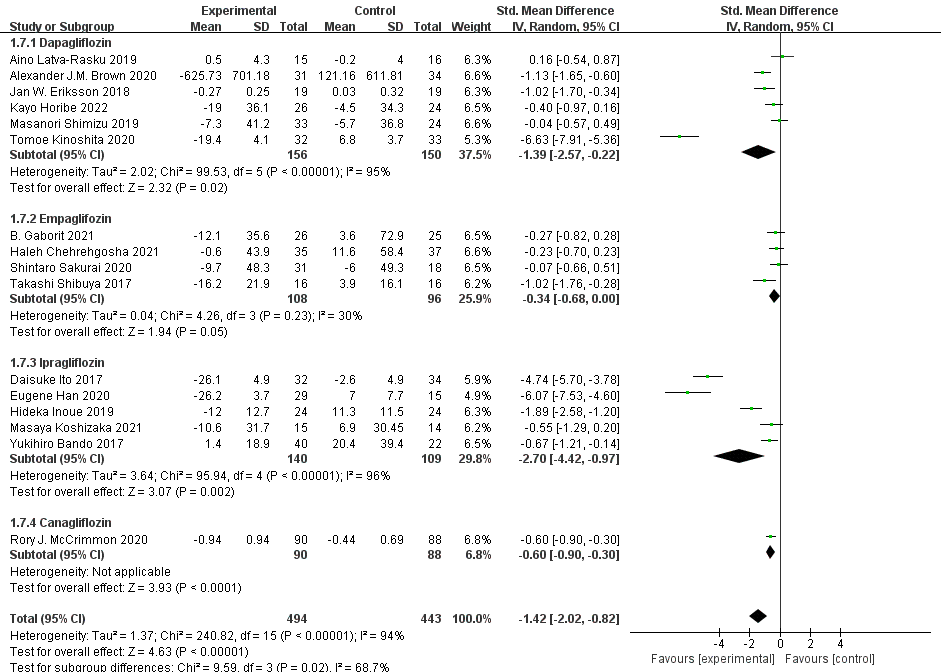

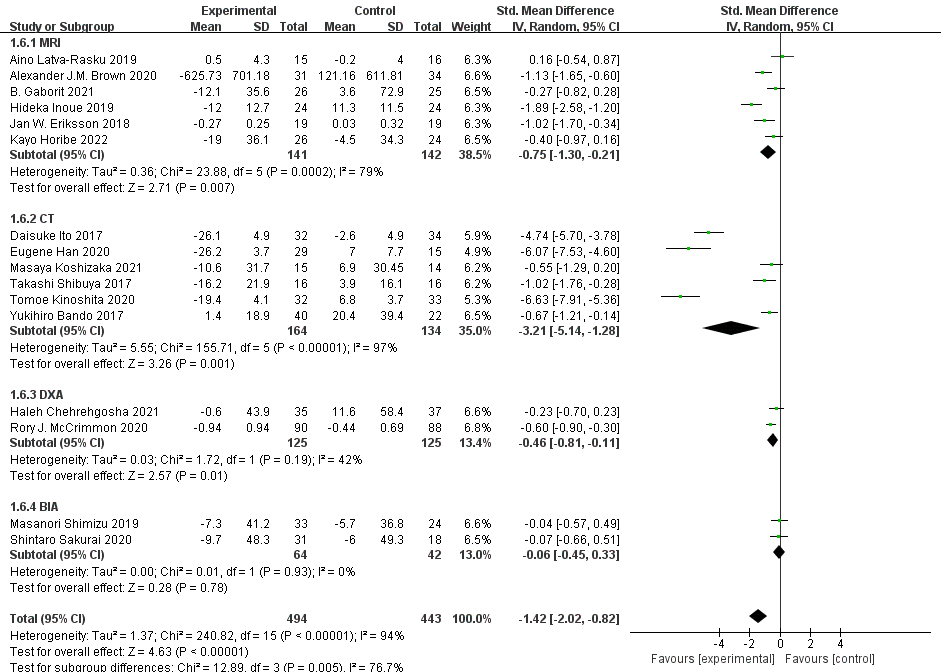

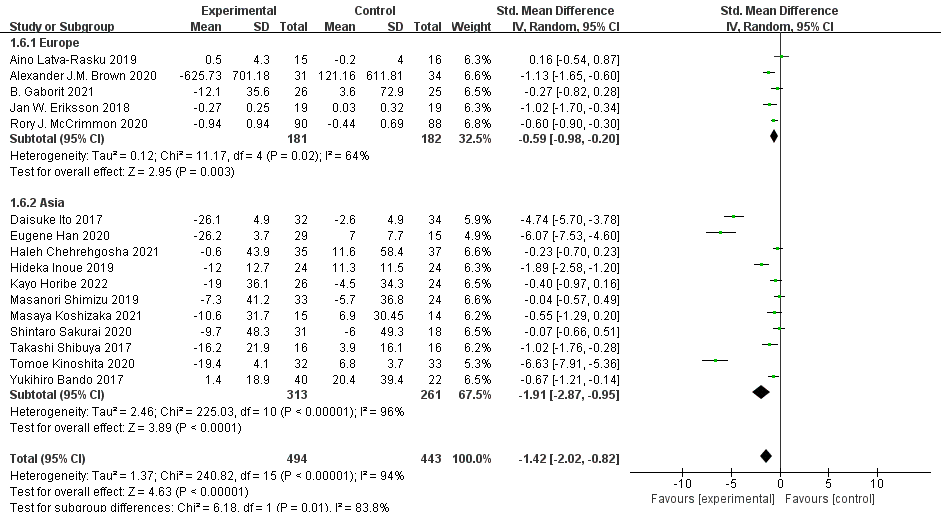


**A**

**B**

**C**

**D**

OAD, oral anti-diabetic drugs

FIGURE S3 (left) Subgroup analyses of SGLT-2 inhibitors efficacy on VAT. (A) Subgroup analysis of VAT based on the measuring method; (B) Subgroup analysis of VAT based on intervention drug; (C) Subgroup analysis of VAT based on the comparator drug; (D)Subgroup analysis of VAT based on country.

FIGURE S4 (right) Subgroup analyses of SGLT-2 inhibitors efficacy on SAT. (A) Subgroup analysis of SAT based on the measuring method; (B) Subgroup analysis of SAT based on intervention drug; (C) Subgroup analysis of SAT based on comparator drug; (D)Subgroup analysis of VAT based on Country.

TABLE S1 Subgroup analysis for liver fat.

| Variables | Group | No. of studies | No. of patients | SMD (95%CI) | *P* | I^2^(%) |
| --- | --- | --- | --- | --- | --- | --- |
| intervention duration | <16 weeks | 2 | 89 | -0.40[−0.82, 0.02] | 0.06 | 0 |
|  | 16≤weeks≤40 | 3 | 173 | -0.91[−1.75, -0.07] | 0.03 | 84 |
| baseline BMI | obesity | 4 | 205 | -0.76[−1.42, -0.10] | 0.02 | 80 |
|  | over weight | 1 | 47 | -0.52[−1.05, 0.02] | 0.06 | - |
| Age | ≥60 years | 1 | 38 | -0.60[−1.25, 0.05] | 0.07 | - |
|  | <60 years | 4 | 224 | -0.73[−1.36, -0.11] | 0.02 | 80 |
| whether with NAFLD | T2DM | 1 | 51 | -0.25[−0.80, 0.30] | 0.37 | - |
|  | T2DM with NAFLD | 4 | 211 | -0.82[−1.43, -0.21] | 0.008 | 77 |

TABLE S2 Subgroup analysis for body weight.

| Variables | Group | No. of studies | No. of patients | SMD (95%CI) | P | I^2^ |
| --- | --- | --- | --- | --- | --- | --- |
| intervention duration | <16 weeks | 4 | 180 | -1.48[−2.30, -0.66] | 0.0004 | 0 |
|  | 16≤weeks≤40 | 8 | 486 | -2.77[−3.61, -1.93] | <0.0001 | 97 |
|  | >40 weeks | 1 | 65 | -3.77[−4.92, -2.62] | <0.0001 | - |
| baseline BMI | obesity | 10 | 577 | -2.63[−3.45, -1.81] | <0.0001 | 95 |
|  | over weight | 3 | 154 | -2.56[−2.78, -2.34] | <0.0001 | 0 |
| Age | ≥60 years | 3 | 151 | -2.77[−3.51, -2.03] | <0.0001 | 56 |
|  | <60 years | 10 | 580 | -2.46[−3.40, -1.53] | <0.0001 | 95 |
| whether with NAFLD | T2DM | 7 | 389 | -2.25[−3.08, -1.43] | <0.0001 | 60 |
|  | T2DM with NAFLD | 6 | 342 | -2.91[−3.91, -1.92] | <0.0001 | 97 |


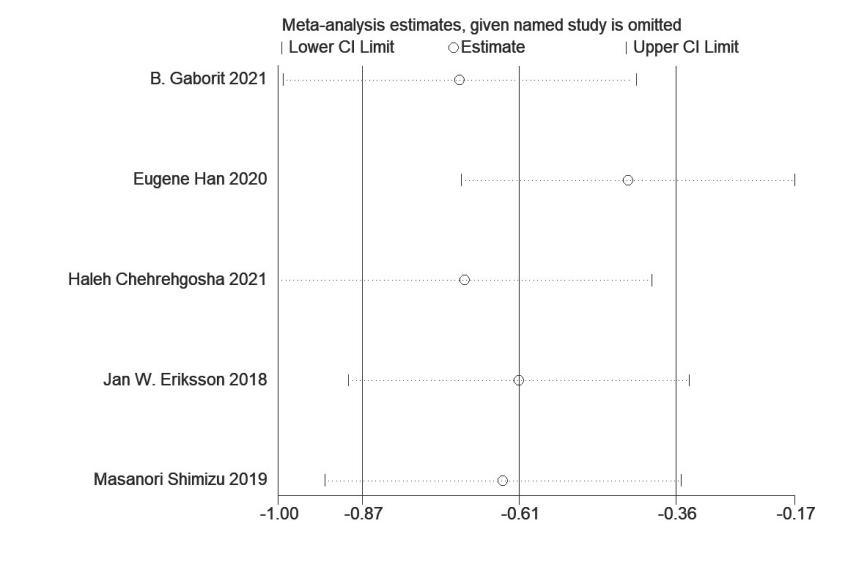


FIGURE S5 Sensitivity analysis of liver fat levels.


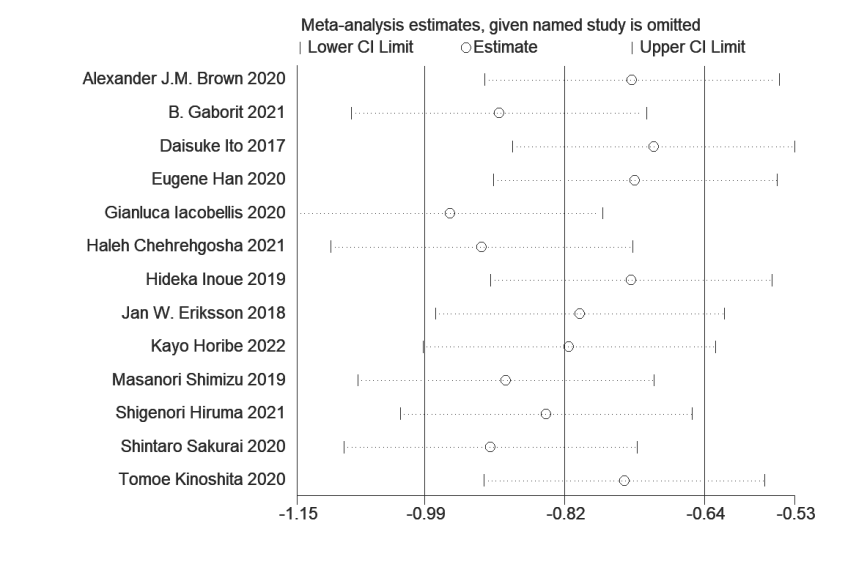


FIGURE S6 Sensitivity analysis of body weight level.
